# Chronic Mucocutaneous Candidiasis in Early Life: Insights Into Immune Mechanisms and Novel Targeted Therapies

**DOI:** 10.3389/fimmu.2020.593289

**Published:** 2020-10-16

**Authors:** Oded Shamriz, Yuval Tal, Aviv Talmon, Amit Nahum

**Affiliations:** ^1^Allergy and Clinical Immunology Unit, Hadassah-Hebrew University Medical Center, Jerusalem, Israel; ^2^The Lautenberg Center for Immunology and Cancer Research, Institute of Medical Research Israel-Canada, Hebrew University-Hadassah Medical School, Jerusalem, Israel; ^3^Pediatrics Department A, Soroka University Medical Center and Faculty of Health Sciences, Ben-Gurion University of the Negev, Beer Sheva, Israel

**Keywords:** CMC, chronic mucocutaneous candidiasis, immune dysregulation, primary immune deficiency, autoimmunity

## Abstract

Children with chronic mucocutaneous candidiasis (CMC) experience recurrent infections with *Candida spp*. Moreover, immune dysregulation in the early life of these patients induces various autoimmune diseases and affects normal growth and development. The adaptive and innate immune system components play a significant role in anti-fungal response. This response is mediated through IL-17 production by T helper cells. Inborn errors in IL-17-mediated pathways or *Candida spp*. sensing molecules are known to cause CMC. In this review, we describe underlying immune mechanisms of monogenic primary immune deficiency disorders known to cause CMC. We will explore insights into current management of these patients and novel available therapies.

## Introduction

Children with chronic mucocutaneous candidiasis (CMC) experience recurrent infections with *Candida spp*. Infections can be mucosal or invasive, and isolated or associated with other infections. CMC can involve the vagina, esophagus, skin, and other organs. Moreover, severe immune dysregulation in the early life of these patients induces various autoimmune diseases and affects normal growth and development. Medical care is complex and usually warrants a combination of systemic anti-fungal and immunosuppressive agents (1–3).

Advances in genetic tests in the recent decade have expanded our knowledge of underlying immune mechanisms in CMC, elucidating an increasing number of newly defined primary immune-deficiency disorders (4). An in-depth characterization of the impaired immune pathways associated with CMC is critical in order to offer treatment tailored to the individual patient.

In this review, we describe monogenic primary immune-deficiency disorders known to cause CMC. Based on insights into underlying immune mechanisms, we explore different targeted therapies currently available or under development for these patients.

## Immune Mechanisms Underlying Monogenic Chronic Mucocutaneous Candidiasis

**Figure 1:**
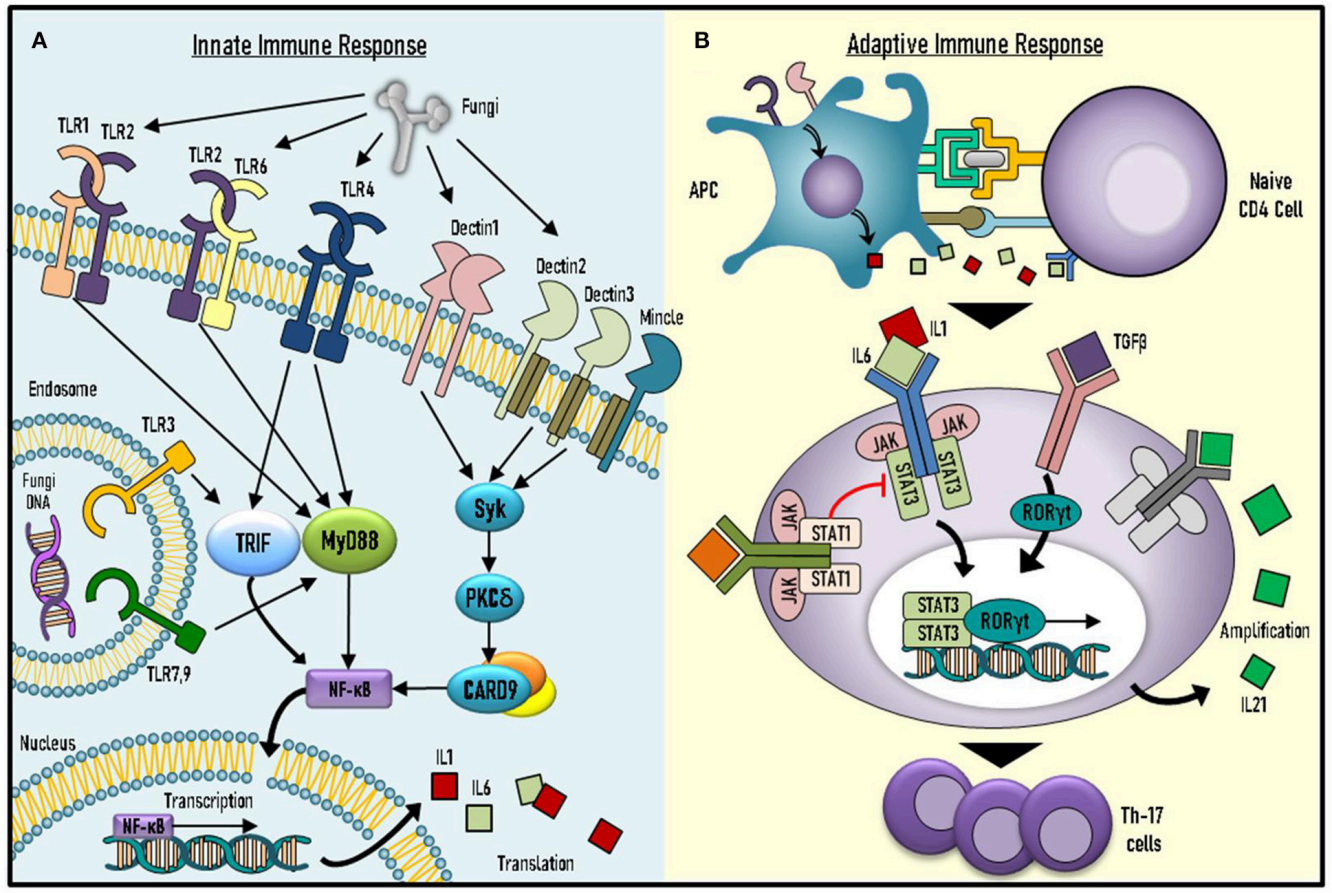
Underlying mechanisms of immune responses against *Candida spp*. **(A)** *Candida spp*. recognition and initial immune response involve key molecules of the innate system. **(B)** Adaptive response against *Candida spp*. includes activation and differentiation of naïve CD4^+^ T cells into effector T helper 17 (Th17) cells. TLR, toll-like receptor; PKC-δ, Protein kinase C-δ; MYD-88, Myeloid differentiation primary response 88; NFκB, Nuclear factor kappa-light-chain-enhancer of activated B cells; IL-Interleukin; APC, Antigen presenting cell; TGF-β, Transforming growth factor beta (TGF-β); JAK-Janus Kinase; STAT, Signal transducer and activator of transcription; RORγT, RAR-related orphan receptor gamma.

The discovery of monogenic causes for CMC has enabled us to expand our knowledge of fundamental immune mechanisms (Figure 1).

Immunity against *Candida* spp. consists of innate and adaptive responses. The innate response involves recognition of pathogen-associated molecular patterns (PAMPs) by pattern recognition receptors (PRRs) found in different cells of the innate immune system, such as monocytes and natural killer (NK) cells (5). Various PRRs are known to induce pro- and anti-inflammatory cytokine production in response to PAMP ligand binding. These PRRs include toll-like receptors (TLRs) 2, 3, 4, 6, and 9, as well as other receptors, such as dectin 1–3 (5). PAMP ligand binding to Dectin-1 leads to signal transduction via adaptor-molecule caspase activation and recruitment domain-containing 9 (CARD9) (5).

The adaptive immune system components also play a significant role in anti-fungal response. This includes pathways mediated by interleukin (IL)-17 and IL-22, which are produced by Th17 cells (6). Indeed, defective fungal sensing by the innate system, as well as abnormalities in IL-17-mediated pathways can induce CMC (Table 1). Impairments in the adaptive response can be further subdivided into decreased IL-17 cytokine production, impaired IL-17-mediated intracellular signaling or increased peripheral neutralization by anti-IL-17 autoantibodies.

**Table 1:** Reported genes associated with chronic mucocutaneous candidiasis.

**Underlying immune mechanism**	**Syndrome**	**Involved gene**	**Inheritance**	**References**
Anti-IL-17 neutralizing autoantibodies	APECED	*AIRE*	AR	(1–3)
IL-17 and IL-17 receptor decreased production	CMC	*IL17F* *IL17RC* *IL17RA*	AR	(4–7)
Defective Th17 differentiation or intracellular signaling	STAT1 gain of function	*STAT1*	AD	(2, 8–18)
	HIES	*STAT3*	AD	(19, 20)
		*DOCK8*	AR	(21)
		*TYK2*	AR	(22)
		*ZNF341*	AR	(23)
		*PGM3*	AR	(24)
		*CARD11*	AD	(25)
	RORγT deficiency	*RORC*	AR	(26)
	ACT1 deficiency	*ACT1*	AR	(27, 28)
	JNK1 deficiency	*MAPK8*	AD	(29)
	MSMD	*IL12*	AR	(30)
		*IL12B*		
		*IL12RB1*		
Decreased *Candida spp*. recognition	CARD9 deficiency	*CARD9*	AR	(31–33)
	Dectin 1 deficiency	*CLEC7A*	AR	(34)

*IL, interleukin; APECED, Autoimmune polyendocrinopathy candidiasis ectodermal dystrophy; AIRE, autoimmune regulator; Th17, T helper 17; DOCK8, dedicator of cytokinesis 8; STAT, Signal transducer and activator of transcription; HIES, hyper IgE syndromes; RORγT, RAR-related orphan receptor gamma; CARD, Caspase recruitment domain-containing protein; PGM3, phosphoglucomutase 3; MSMD, Mendelian Susceptibility to Mycobacterial Diseases; TYK2, tyrosine-protein kinase 2; JNK1, c-Jun N-terminal kinase 1; AD, autosomal dominant; AR, autosomal recessive; CLEC7A, C-Type Lectin Domain Containing 7A*.

### Production of Neutralizing Autoantibodies Against IL-17 and IL-22

T cell development in the thymus includes clonal deletion of self-reactive T cells. This is achieved by the introduction of self-antigens to naive T cells by medullary thymic epithelial (mTEC) and dendritic cells. mTECs express autoimmune regulator (AIRE), an important facilitator of self-antigen gene expression (7).

AIRE deficiency is characterized by loss of self-tolerance and the presence of autoreactive T cells and multiple severe autoimmune diseases. *AIRE* loss-of-function (LOF) induces autoimmune polyendocrinopathy-candidiasis-ectodermal dystrophy (APECED). APECED is characterized by a classical triad of CMC, hypoparathyroidism, and Addison's disease (8), but other systems can be affected by autoimmunity in APECED, which can induce type 1 diabetes, hypothyroidism, hypogonadism, vitiligo, and various other autoimmune diseases (8). CMC in APECED patients is explained by decreased IL-17 and IL-22 cytokine serum levels, with corresponding increased titers of anti-IL-17 and anti-IL-22 neutralizing autoantibodies (9, 10). Thus, anti-IL-17/22 autoantibody production in APECED demonstrates the important association between immune dysregulation and CMC susceptibility.

### Inborn Errors in IL-17 Production or IL-17 Receptor Surface Expression

IL-17R-mediated signaling has been shown in murine models to be essential in the immune response against *Candida spp*. (11, 12). In 2011, a single patient was reported to have a homozygous c.850C>T mutation in *IL-17RA* that caused reduced surface expression of IL-17RA on peripheral blood mononuclear cells (PBMC), reduced lymphocyte response to IL-17A/F stimuli, and increased susceptibility to *Candida spp*. infections (13). Two other patients with CMC had impaired IL-17F cytokine production due to *IL-17F* gene mutation (13, 14). Since then, several cohorts of CMC patients with IL-17R deficiencies have been reported, including 21 patients from 12 unrelated families with *IL-17RA* deficiency (15) and three patients with *IL-17RC* deficiency (16).

### Defective Th17 Differentiation or Intracellular Signaling

Antigen-presenting cells produce IL-6, IL1-β, and IL-23, as well as activate transforming growth factor (TGF)-β in response to fungal infections. These cytokines bind to naïve CD4^+^ T cells and trigger STAT3 followed by RAR-related orphan receptor (ROR)γT-mediated transcription. RORγT enhances production of IL-17A, IL-17F, and IL-21 by lymphocytes, through which they differentiate into Th17 cells. In turn, IL-21 further self-amplifies Th17-mediated immune responses (17, 18).

#### STAT1 Gain of Function

Inborn errors in any of the key players in Th17 differentiation can result in CMC. STAT1 is such a key component as was demonstrated from the study of autosomal dominant (AD) gain-of-function (GOF) mutations. *STAT1* mutations are probably the most common cause of monogenic CMC. These patients present with a wide clinical spectrum of immune dysregulation and increased susceptibility to bacterial, viral and fungal infections (19). Delayed dephosphorylation of STAT1 in these patients impairs the function of IL-6 and IL-21, thus decreasing STAT3-dependent differentiation of naïve CD4^+^ T cells into Th17 cells (20). Of note, a recent report suggests that some *STAT1* GOF mutations may cause STAT1 levels to be high, although phosphorylation is normal (21). Disease severity appears to vary according to the mutation. For example, patients with the T385M mutation are somewhat phenotypically different from others. The T385M clinical spectrum consists of chronic candidiasis, recurrent severe invasive infections with bacterial pathogens, severe viral infections such as cytomegalovirus and John Cunningham virus and, last but not least, severe autoimmune phenomena reminiscent of a combined immunodeficiency disease. These patients show progressive loss of T and B cell function (22).

#### Hyper IgE Syndromes

Another striking example of impaired Th17 differentiation is *STAT3* LOF mutations known to cause autosomal dominant hyper immunoglobulin E syndrome (AD-HIES). These patients have severe eczema, skin abscesses, staphylococcal infections, and decreased or absent Th17 cells, resulting in increased susceptibility to *Candida* infections (23, 24). Markedly increased IgE levels and eosinophilia are indicative of immune dysregulation in these patients (25). *STAT3* LOF patients are distinctive by their non-immunologic features, which include dysmorphic facial features, retained primary teeth, vascular aneurysms, scoliosis, osteoporosis, and other musculoskeletal manifestations (26).

Autosomal recessive (AR) HIES is caused by mutations in *dedicator of cytokinesis* (*DOCK*) *8, ZNF341*, and *tyrosine kinase (TYK)2*. DOCK8 plays an important role in T cell activation and proliferation via its role in T cell cytoskeleton and actin reorganization. *DOCK8* mutation results in abnormal Th17 polarization and function (27). Clinical manifestations include an immune dysregulation phenotype consisting of allergic disorders, such as atopic dermatitis and food allergies, as well as increased susceptibility to staphylococcal, sino-pulmonary and viral infections (26).

Other gene mutations causing AR-HIES have been reported in *ZNF341*. This factor regulates the transcription of *STAT3*, therefore patients with ZNF341 deficiencies are clinically similar to HIES with *STAT3* LOF. They are reported to have low levels of STAT3, reduced numbers of Th17 cells, and high risk for CMC (28). *TYK2*, a JAK family member, is critical for normal IL-12 and type I IFN expression. Mutation of *TYK2* can also cause AR-HIES. A patient with a homozygous TYK2 mutation was reported to have increased susceptibility to viral infections due to an impaired IFN-mediated response, and increased risk for fungal infections most probably due to defective IL-12/IL-23-mediated responses (29).

In addition, we should mention phosphoglucomutase (*PGM)3 and CARD11 deficiencies, both* reported in some studies to induce CMC and HIES. PGM3 deficiency is an AR-HIES disorder characterized by glycosylation defects that have multi-systemic manifestations including a neurodegenerative course. Sassi et al. reported occurrence of CMC in four out of nine patients (30), whereas Zhang et al. and Stray-Pederson et al. did not describe such findings (31, 32). LOF mutations in CARD11 were associated with severe atopy and immune dysregulation (33). In both disorders, it appears that Th17 cells are present, rather than absent. Therefore, the defect is probably functional and in the context of global T cell defects.

#### IL-12/IL-12 Receptor Pathway

Inborn errors in IL-12-mediated pathways are known to play a major cause for Mendelian susceptibility to mycobacterial disease (MSMD), increasing the risk for mycobacterial and viral infections. Interestingly, impaired defective IL-12 or IL-12R may underlie abnormal IL-23-mediated signaling, thus also exposing these patients to risk of developing CMC (13). Defective IL-23- and IL-12-mediated pathways were previously reported in patients with IL-23R and IL-12Rβ2 deficiencies, respectively. Impaired signaling in these patients induced MSMD; however no CMC was observed (34).

#### RORC, ACT1, and MAPK8 Mutations

STAT3 induces RORγT transcription, which leads to Th17 differentiation. AR mutations in RORγT have been demonstrated to decrease Th17 cell counts and result in CMC. Interestingly, these patients also presented with increased susceptibility to mycobacterial infections due to impaired interferon (IFN)-γ-mediated immunity, which also requires RORγT (35).

Regarding the IL-17-mediated pathway, one should also remember other proteins downstream. ACT1 is an intracellular adaptor protein in the IL-17-mediated signaling pathway. Several human mutations in *ACT1* are known to impair Th17 function and induce CMC (36, 37). *Staphylococcus aureus* blepharitis (37) and recurrent pneumonia (36) were also noted in these patients, who display characteristics of primary immune deficiency with dysregulation.

Finally, we should also mention mutations in *MAPK8*. AD *MAPK8* mutations resulting in c-Jun N-terminal kinase 1 (JNK1) deficiencies were previously reported to induce CMC. Impaired Th17 differentiation and decreased responses to IL-17A and IL-17F stimuli were shown. Interestingly, JNK1-deficient patients with CMC were also found to have a novel connective tissue disease, thus distinguishing mutant *MAPK8* from other monogenic inducers (38).

### Decreased Recognition of Candida Infections

The innate response against *Candida spp*. is complex. Recognition of fungal PAMPs by PRR is critical for *Candida spp*. sensing, as is the Dectin-1–Syk–CARD9 signaling pathway. Biallelic mutations in CARD9 are reported to induce CMC and general increased susceptibility to fungal infections (39–45). In comparison with IL-17-associated inborn errors, CARD9 deficiency is thought to induce a more severe and invasive candidiasis, affecting various tissues including even the central nervous system (CNS) (46).

Dectin-1 deficiency has also been shown to induce reduced recognition of β-glucans with increased susceptibility to *Candida spp*. infections. However, an important feature of this disorder is the lack of susceptibility to other infections, which defines it as an isolated CMC (47). Impairment of the Dectin-1–Syk–CARD9 pathway also affects the differentiation of CD4^+^ naïve T cells into Th17 cells, thereby interfering with the adaptive immune response to *Candida spp*. (6). Indeed, Tyr238X mutation in dectin-1 was previously described to cause CMC and onychomycosis phenotypes, as well as decreased IL-17 levels. However, phagocytosis and killing of *Candida spp*. in these patients were intact (47). Although dectin-1 deficiency is not included in International Union of Immunological Societies (IUIS) 2019 classification (4), the Tyr238X mutation can be found in gnomAD^1^.

## Current Management of Monogenic Chronic Mucocutaneous Candidiasis

Current management of CMC consists mainly of prophylactic anti-fungal agents, such as fluconazole (1). However, other therapeutic modalities are currently available. Granulocyte-macrophage colony-stimulating factor (GM-CSF) production by PBMC is suggested to be reduced in CARD9-deficient patients. A patient with a hypomorphic CARD9 mutation presenting with CNS candidiasis was found to achieve clinical remission after GM-CSF administration (46), and GM-CSF has been found to be effective in other patients with CARD9 deficiency (48).

Histone deacetylase (HDAC) inhibitors were also examined in the management of CMC, especially in patients with STAT1 GOF mutations. Inhibition of histone acetylation is thought to affect the adaptive and innate immune systems. Indeed, HDAC inhibitors were found to rescue STAT3-mediated pathways in STAT1 GOF patients (49). Moreover, *in-vitro* treatment with HDAC inhibitors resulted in increased IL-22 production in response to *Candida spp*. (49).

Hematopoietic stem cell transplantations (HSCT) have some efficacy in CMC. For example, in two patients with CARD9 deficiency, HSCT from haploidentical and fully matched donors was successful, although a second HSCT was required in the first patient. Complete clinical resolution of fungal infections was noted in both patients (45). There are reports of successful HSCT in *STAT1* GOF patients as well, with complete resolution of immune dysregulation and rescue of Th17 differentiation and function (50). However, the results of HSCT in *STAT1* GOF are generally not favorable, with high rates of secondary graft failure (51).

Targeted immunotherapies for CMC-inducing inborn errors are therefore warranted. Ruxolitinib, a Janus kinase (JAK)1/2 inhibitor, is reportedly effective in *STAT1* GOF. Ruxolitinib treatment of a *STAT1* GOF child presenting with a clinical picture of CMC and autoimmune cytopenia was shown to directly intervene with the impaired immune pathways. It improved Th17 differentiation, decreased Th1-mediated responses, and attenuated CMC and immune dysregulation (52). Another study found that ruxolitinib in *STAT1* GOF patients can rescue NK cell maturation. Moreover, it was effective in restoring perforin expression on NK cells, thus rescuing NK cytotoxic function (53). Other reports of children with *STAT1* GOF mutations have confirmed the efficacy and safety of ruxolitinib in this disorder (54–56).

## Conclusions

Current advances in next-generation sequencing have revealed various monogenic inducers of CMC. Understanding the impaired immune pathways involved in CMC is critical in the management of these patients. CMC is strongly associated with immune dysregulation and autoimmunity in early childhood. Therefore, a joint collaboration between immunologists, endocrinologists, and infectious disease and other specialists is needed in order to offer a personally tailored, effective, treatment to these patients.

## Author Contributions

OS study design, review of the literature, and manuscript writing. YT and AN study supervision and manuscript revisions. AT immune consultation and manuscript revision. All authors agree to be accountable for the content of the work.

## Conflict of Interest

The authors declare that the research was conducted in the absence of any commercial or financial relationships that could be construed as a potential conflict of interest.

## Footnotes

^1^https://gnomad.broadinstitute.org/variant/12-10271087-A-C?dataset=gnomad_r2_1
